# Determination of HE4 in pleural fluid and ratio: considerations and diagnostic performance for malignant pleural effusion

**DOI:** 10.1515/almed-2025-0175

**Published:** 2026-04-07

**Authors:** Elisa Nuez-Zaragoza, Indira Bhambi-Blanco, Isabel Aparicio-Calvente, Mònica Vidal-Pla, M. Rosa Escoda-Giralt, Joana Gallardo-Campos, Joan C. Ferreres, Luis Frisancho, Laia Mas-Maresma, Patricia Aguilera-Fernández, Sonia Marco-Continente, Marina Sierra-Boada, Pablo Andreu-Cobo, Miquel Gallego, Vicente Aguadero, Jaume Trapé

**Affiliations:** Clinical Laboratory Department, Parc Taulí University Hospital, Institut d’Investigació i Innovació Parc Taulí (I3PT-CERCA), Universitat Autònoma de Barcelona, Sabadell, Barcelona, Spain; Department of Pathology, Parc Taulí University Hospital, Institut d’Investigació i Innovació Parc Taulí (I3PT-CERCA), Universitat Autònoma de Barcelona, Sabadell, Barcelona, Spain; Department of Gastroenterology, Parc Taulí University Hospital, Institut d’Investigació i Innovació Parc Taulí (I3PT-CERCA), Universitat Autònoma de Barcelona, Sabadell, Barcelona, Spain; Department of Internal Medicine, Parc Taulí University Hospital, Institut d’Investigació i Innovació Parc Taulí (I3PT-CERCA), Universitat Autònoma de Barcelona, Sabadell, Barcelona, Spain; Intensive Care Unit, Parc Taulí University Hospital, Institut d’Investigació i Innovació Parc Taulí (I3PT-CERCA), Universitat Autònoma de Barcelona, Sabadell, Barcelona, Spain; Department of Oncology, Parc Taulí University Hospital, Institut d’Investigació i Innovació Parc Taulí (I3PT-CERCA), Universitat Autònoma de Barcelona, Sabadell, Barcelona, Spain; Department of Pulmonology, Parc Taulí University Hospital, Institut d’Investigació i Innovació Parc Taulí (I3PT-CERCA), Universitat Autònoma de Barcelona, Sabadell, Barcelona, Spain; Department of Biochemistry, CLILAB, Sant Joan Despí-Moisès Broggi Hospital, Sant Joan Despí, Spain; Department of Biochemistry, ALTHAIA Xarxa Assitencial Universitària de Manresa, Universitat de Vic – Universitat Central de Catalunya, School of Medicine, Manresa, Spain

**Keywords:** HE4, pleural fluid, HE4 ratio, malignant pleural effusion

## Abstract

**Objectives:**

The objectives of this study included assessing the diagnostic performance of HE4 in malignant pleural effusions (MPEs) and identifying the benign etiologies associated with higher HE4 concentrations.

**Methods:**

The study involved patients with pleural effusion (PE) treated at Part Taulí University Hospital. PE and serum samples were collected and analyzed. Diagnosis of MPE was established by the presence of a positive cytology and/or positive pleural biopsy. HE4 concentrations in LP and serum – among other parameters – were measured on an ECLIA-Cobas e801 (Roche Diagnostics) analyzer. Subsequently, pleural fluid to serum HE4 ratio (PF/serum HE4 ratio) was calculated. Patients were assigned to different groups according to final diagnosis, transudate vs. exudate, MPE etiology and estimated glomerular filtration rate (eGFR). An evaluation was performed of the diagnostic performance of HE4 for MPE.

**Results:**

A total of 253 PF and serum samples were included. In patients with benign pleural effusion (BPE), transudates contained higher levels of HE4, as compared to exudates. The highest PF-HE4 concentrations and ratios were observed in MPEs, especially in patients with non-small cell lung cancer + ovarian cancer. The cut-off values established showed a sensitivity of 16.7 % (PF-HE4) and 12.1 % (ratio), with a 100 % specificity, respectively. Following cut-off adjustment, higher sensitivity values were observed in patients with an eGFR ≥ 30 mL/min/1.73 m^2^.

**Conclusions:**

Elevated HE4 concentrations may support MPE diagnosis and predict histology. Transudates contain elevated levels of HE4 and lower PF/serum-HE4 ratios. In the investigation of solid neoplasms, different serum HE4 cut-off values should be used for patients with PE, heart failure, cirrhosis or renal insufficiency.

## Introduction

Pleural effusion (PE) is the build-up of fluid within the pleural cavity as a result of the presence of different diseases [1]. Pleural fluid biochemical and cytological analyses are essential for determining PE etiology [2], 3], enabling the classification of effusions as transudative or exudative, which is the first diagnostic step [2], [3], [4]. Transudates are primarily associated with heart failure and liver cirrhosis, whereas exudates are most commonly related to cancer, pneumonia, tuberculosis or viral pleuro-pericarditis, among other conditions [2], 3]. MPE is the most common cause of exudative pleural effusion, accounting for 95 % of exudates [5]. The remaining 5 % correspond to transudates, including paramalignant PEs, where no tumor cells are present in the pleura, but other processes regulating serous fluid formation and/or absorption are involved [6].

The prevalence of malignant pleural effusion (MPE) is 15–35 % [2]. MPE may be primary (pleural tumors such as mesothelioma) or secondary, when caused by the infiltration of another primary neoplasm, accounting in many cases for the first clinical sign of undiagnosed cancer [2], 7]. MPE is a common complication of lung cancer (most commonly, adenocarcinoma), breast cancer, and hematological malignancies, among other oncological conditions [2], 5], 8]. MPE is an indicator of advanced tumor stage and poor prognosis, with an average survival rate <1 year [2], 5], 8], 9].

The gold-standard method for MPE diagnosis is cytological analysis [10], with a high specificity but inconsistent sensitivity, ranging from 40 to 86 % as reported in the literature [5], 8], [10], [11], [12]. Confirmation by subsequent cytological or invasive procedures such as pleural biopsy is required [2], 5], 8], 10], 12].

The use of non-invasive markers for the diagnosis of MPE is essential and complements cytological analysis. Non-invasive markers include human epididymis protein 4 (HE4), which has been validated for the diagnosis and monitoring of ovarian cancer. However, this biomarker has been documented to be overexpressed in other types of cancer, such as lung cancer, endometrial cancer, breast adenocarcinoma, and pancreatic tumors, among other types of malignancies [13], [14], [15], [16]. Several meta-analyses have underlined the role of this serum parameter as a potential diagnostic marker in lung cancer [14]. Hence, HE4 overexpression is related to advanced stage and poorer survival rates [13]. However, the results reported in the different studies are inconsistent owing to the presence of other confounding factors influencing HE4 levels, like kidney function [7], [13], [14], [15], [16].

In contrast, the literature on HE4 levels in PF is limited. Higher levels of HE4 have been documented in MPE cases, as compared to benign pleural effusion (BPE), especially in ovarian and lung cancer [7], 17], 18]. However, variability is observed in the reported cut-off values and diagnostic performance of this marker [7], [17], [18], [19].

The influence of kidney function on HE4 levels in serum and biological fluids has been previously examined, with increased levels observed in patients with renal insufficiency (RI) [7], 20]. The most widely used method for interpreting tumor marker concentrations and avoiding false positives in MPE diagnosis related to RI is by calculating PF/serum HE4 ratio [6]. Notably, the HE4 ratio has been investigated in only a study [19], where significant differences were not documented between MPE and BPE.

The purpose of this study was to assess the diagnostic performance of HE4 concentrations in PF and serum, along with the PF/serum HE4 ratio for MPE in our cohort of patients. The secondary objective was to identify the benign etiologies associated with the highest HE4 concentrations in PF and serum and the highest PF/serum HE4 ratios.

## Materials and methods

### Study design and participants

A prospective, experimental, single-center study was conducted in Parc Taulí University Hospital. This study was approved by the local Research Ethics Committee (code 2021/5058). Informed consent was obtained from all participants.

The sample included 341 adult inpatients and outpatients with PE treated in our hospital between May 2021 and November 2022 in the following departments: Emergency Department, Internal Medicine, Intensive Care Unit, Gastroenterology, Pulmonology and Oncology Department. The totality of patients had been diagnosed with PE and underwent a diagnostic thoracentesis.

The clinical samples used in this study were the same as those employed in a previous study conducted by our research group [21]. The exclusion criteria used in this study were: ICU admission, hemodialysis and absence of a conclusive final diagnosis six months after sample collection.

### Pleural fluid and serum analysis

#### Sample collection

Following sample extraction, PF and serum samples were sent to the Hospital Clinical Laboratory. All samples were collected for routine diagnostic purposes. No additional samples were extracted for the purposes of this study.

PF was collected in sterile, additive-free containers and subsequently divided into two aliquotes. An aliquote was sent to the Clinical Laboratory for cell counting, differentiation and biochemical analysis. The other aliquote, containing ≥10 mL of fluid, was sent to the Department of Pathology for cytological analysis, following anonymization. When the sample was directly sent for cytological analysis after collection, an additional sample was not sent from the Clinical Laboratory. In the two laboratories, the sample was immediately processed after collection.

#### Cell counting and differentiation

Cell counting was performed using the biological fluid mode of the Sysmex XN-10 analyzer (Sysmex, Kobe, Japan). The following parameters were analyzed by fluorescence flow cytometry: white blood cells (WBC); polymorphonuclear percentage (PMN%) (neutrophils, eosinophils and basophils); and mononuclear cell percentage (MN%) (lymphocytes, macrophages and plasma cells). Red blood cell (RBC) count was obtained through electric impedance.

#### Cytological study

Cytological analysis involved Papanicolau and Diff-Quick staining for an initial morphological analysis, followed by immunohistochemistry techniques to confirm MPE, when abnormal cells suggestive of malignancy were observed. Diagnosis of MPE was established by cytological analysis and/or a positive pleural biopsy (by pleuroscopy or thoracoscopy). Then, patients were classified into two groups: MPE and BPE. Final diagnosis was confirmed by reviewing the patient’s medical record six months after sample collection.

#### Biochemical analysis

Following cytological analysis, PF was centrifuged at 1,500 rpm for 5 min. The supernatant was used for biochemical analysis and HE4 quantification. Blood samples were centrifuged at 3,500 rpm for 10 min to separate serum.

Glucose (hexokinase method, Ref. 08057800190); lactate dehydrogenase (LDH) (lactate to pyruvate method, Ref. 08057958190); and total proteins (biuret method, Ref. 08058652190) were measured in PF and serum using the Cobas c501 analyzer (Roche Diagnostics, Switzerland). Adenosine deaminase (ADA) was determined in PF using the BioSystems reagent (Ref. 03501950001) on the same analyzer.

PF/serum LDH ratio and PF/serum protein ratio were calculated to identify samples as transudative or exudative according to Light’s criteria [22]. Hence, a PE was classified as an exudate when it met one or several of the following criteria: PF/serum protein ratio >0.5; PF/serum LDH ratio >0.6; or PF LDH level >2/3 the upper limit of normality for LDH in serum.

Creatine was quantififed in serum (Jaffé method, Ref. 08057532190) on a Cobas c501 analyzer, whereas estimated glomerular filtration rate (eGFR) was calculated using the CKD-EPI formula [23].

Following collection, samples were stored at −80° for later HE4 quantification in PF and serum. Then, the PF/serum HE4 ratio was calculated (PF/serum HE4 ratio). Analyses were performed on an electrochemiluminescence immunoassay-based Cobas e801 (Roche Diagnostics, Switzerland) analyzer (Ref. 07027478214).

### Statistical analysis

The Kolmogorov–Smirnov test was used to check the normality of distribution of quantitative variables. In the case of normality, continuous variables were expressed as mean and standard deviation, whereas non-parametric variables were presented as median and interquartile ranges (ICR, P25–P75). Based on final diagnosis, patients were categorized into two groups: BPE and MPE. The BPE group was subdivided into two groups: transudates and exudates. The MPE group was subdivided into three groups: non-small cell lung cancer (NSCLC) with concomitant ovarian cancer; small cell lung cancer (SCLC) and other malignancies. The group with NSCLC with concurrent ovarian cancer was established based on the evidence available in the literature on HE4 overexpression in this group of patients, as compared to other malignancies.

A comparative analysis was performed to identify differences across groups: BPE vs. MPE total; transudative BPE vs. exudative BPE; and NSCLC + ovarian cancer vs. other malignancies. Differences between SCLC and NSCLC were not assessed due to the small sample of SCLC patients included. Differences in the BPE group according to the eGFR were also examined (eGFR ≥30 mL/min/1.73 m vs. <30 mL/min/1.73 m^2^). Prior to this step, the distribution of HE4 concentrations in PF, serum, along with HE4 ratio according to patients’ eGFR were studied. Then, samples were divided into four groups: ≥60 mL/min/1.73 m^2^, 45–60 mL/min/1.73 m^2^, 30–45 mL/min/1.73 m^2^ and <30 mL/min/1.73 m^2^.

For independent samples, differences between means were assessed using Student’s t test, whereas Mann–Whitney U test was used when at least one of the variables did not follow normal distribution. A p-value <0.05 was considered statistically significant.

The diagnostic performance of HE4 for MPE was assessed in the different samples by calculating the area under the receiver operating characteristic (ROC) curve (AUC). Differences in AUC were compared using the DeLong test. Sensitivity and positive and negative predictive values were additionally calculated for the cut-off values corresponding to maximum specificity.

Statistical analyses were carried out using the IBM^®^ SPSS^®^ Statistics software package for Windows, version 20 (IBM Corporation, Armonk, New York, USA).

## Results

**Figure 1:**
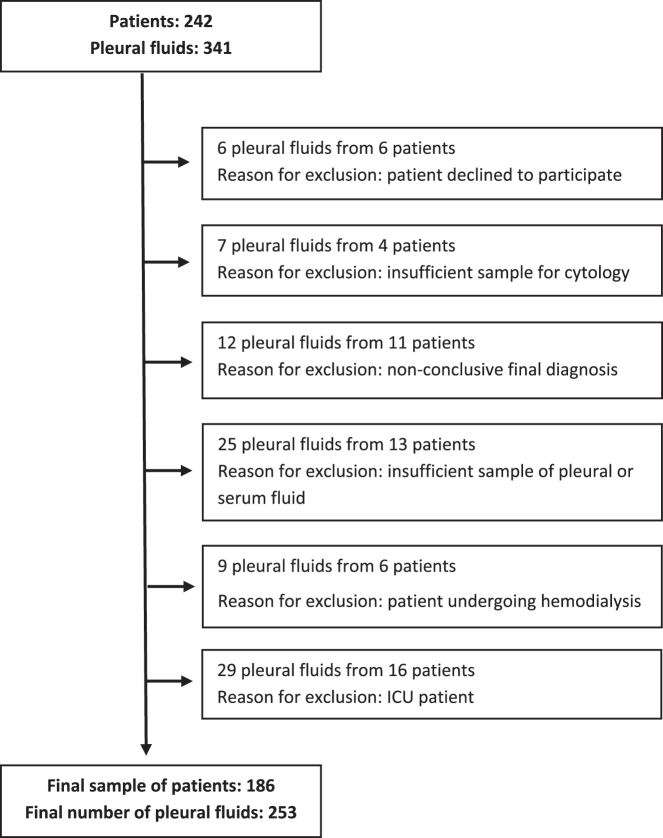
Patient flowchart and exclusion criteria.

The study included 253 samples of PF and serum from 186 patients (74 women and 112 men). Figure 1 depicts patient flowchart by showing exclusion criteria.

Causes of PE are summarized in Table 1. A total of 188 BPE cases (74.31 %) and 65 MPE cases were identified. Lung cancer was the most common malignancy (32 cases); 27 cases were NSCLC and 5 SCLC.

**Table 1:** Pleural effusion etiologies.

Etiology		n (%)
Neoplasm		65 (25.69 %)
Lung cancer	32 (49.23 %)	
Hematological neoplasms	8 (12.31 %)	
Mesothelioma	7 (10.77 %)	
Breast cancer	5 (7.69 %)	
Pancreatic cancer	3 (4.62 %)	
Esophageal cancer	3 (4.62 %)	
Colon cancer	3 (4.62 %)	
Ovarian cancer	2 (3.08 %)	
Prostate cancer	1 (1.54 %)	
Neuroendocrine cancer	1 (1.54 %)	
Heart failure		52 (20.55 %)
Parapneumonic and empyema		41 (16.21 %)
Cirrhosis		27 (10.67 %)
Unknown etiology		14 (5.53 %)
Post-surgery		9 (3.56 %)
Post-traumatic		9 (3.56 %)
Tuberculosis		9 (3.56 %)
Pulmonary thromboembolism		5 (1.98 %)
Liver abscesses and cholangitis		4 (1.58 %)
Autoimmune		4 (1.58 %)
Pleuropericarditis		4 (1.58 %)
Asbestosis		3 (1.19 %)
Pleuritis fibrosa		3 (1.19 %)
Paramalignant		2 (0.79 %)
Pancreatitis		1 (0.40 %)
Atelectasis		1 (0.40 %)
Total		253

n, number of samples.

The results and the comparison of the variables are shown in Table 2. Significant differences were observed in PF HE4 concentration and HE4 ratio between the total BPE and total MPE group. No significant differences were noted in serum. Within the BPE group, transudates revealed lower eGFR and higher HE4 concentrations in PF and serum, as compared to exudates. HE4 ratio was also lower in transudates, although differences were not statistically significant.

**Table 2:** Differences in study variables across study groups.

		BPE			MPE			
	Total	Total BPE	Transudative BPE	Exudative BPE	Total MPE	NSCLC + ovarian	SCLC	Other neoplasms
n	253	188	45	143	65	29	5	31
Qualitative variables								
Sex, F/M	107/146	80/108	23/22	57/86	27/38	13/16	2/3	12/19
Type of effusion (exudate/transudate)	206/47	143/45	0/45	143/0	63/2	28/1	5/0	30/1
Cytology (positive/negative)	48/205	0/188	0/45	0/143	48/17	23/6	3/2	22/9
Quantitative variables								
Age, years	72.03 ± 13.93	72.8 ± 14.33	75.87 ± 10.16	71.84 ± 15.32	69.8 ± 12.54	69.34 ± 13.15	64 (4)	71.29 ± 12.76
WBCs, cells/µL	681 (2,100)	541 (1,737.25)	164 (149)	891 (2,202)^a^	1,626 (2,858)^b^	1,910.55 ± 1,880.82	1,171 (2,327)	1,846 (3,386)
RBCs, cells/µL	5200 (31,900)	4,000 (23,837.5)	500 (1,200)	8,100 (43,550)^a^	14,100 (53,300)^b^	20,300 (34,000)	6,300 (400)	28,100 (119,700)
PMN, %	23 (39)	25 (40)	19.56 ± 13.9	30 (44.5)^a^	13 (33)^b^	27.14 ± 27.74	13 (38)	12 (26.5)
MN, %	78 (40)	75 (39.25)	80.29 ± 13.82	70 (44.5)^a^	87 (33)^b^	72.59 ± 27.51	87 (38)	88 (26.5)
Glucose PF, g/dL	112 (58)	116.5 (61)	150.89 ± 41.59	113 (55)^a^	95.63 ± 50.48	94.83 ± 53.2	113 (63)	90.71 ± 45.68
ADA PF, U/L	22.1 (13.2)	22 (14.82)	12.8 (5.9)	25.6 (12.65)^a^	22.8 (7.7)	21.2 (7.3)	14.4 (6.9)	27.24 ± 12.13
PF total proteins, g/L	39 (16)	37 (19)	21 (11)	42 (2.5)^a^	40 (12)^b^	43.17 ± 9.25^c^	40 (6)	36.42 ± 11.73
PF LDH, U/L	207 (353.5)	160.4 (304.33)	71 (27.6)	225 (448.8)^a^	333 (447.2)^b^	300 (411.4)	325 (3,683)	411.7 (491.7)
Serum total proteins, g/L	64.6 (10)	64.7 (9.4)	62.2 (6)	65 (9.65)^a^	64 (12)	66 (8.2)	62.1 (7)	63.67 ± 8.39
Serum LDH, U/L	201 (94.32)	195 (87.8)	221.8 (84.1)	190 (82.85)^a^	225 (116.8)^b^	220.13 (78.47)^c^	256 (1,304)	248.2 (148.2)
Serum creatinine, mg/dL	0.91 (0.68)	0.95 (0.78)	1.37 (1)	0.91 (0.67)^a^	0.88 (0.4)^b^	0.97 (0.33)	0.57 (0.11)	0.91 ± 0.3
eGFR, mL/min/1.73 m^2^	69.54 ± 30.32	66.45 ± 31.89	53.22 ± 31.36	70.62 ± 31^a^	78.46 ± 23.25^b^	74.96 (21.76)	101.62 (10.8)	71.29 ± 32.5
PF HE4, pmol/L	673 (590)	658.5 (480)	775 (451)	611 (455)^a^	805 (1,172)^b^	1,666 (2,299)^c^	495 (169)	648 (592.5)
Serum HE4, pmol/L	173 (221)	189 (213.75)	273 (263)	161 (186.5)^a^	143 (207.7)	234 (377)^c^	106 (47.7)	136 (97.85)
HE4 ratio	3.36 (3.51)	3.09 (3.54)	2.78 (3.16)	3.13 (3.6)	4.07 (4.82)^b^	4.44 (11.19)	4.12 (0.11)	3.63 (3.54)

ADA, adenosine deaminase; eGFR, estimated glomerular filtrate; SCLC, small cell lung cancer; NSCLC, non-small cell lung cancer; BPE, benign pleural effusion; MPE, malignant pleural effusion; F, female; LDH, lactate dehydrogenase; PF, pleural fluid; M, male; MN, mononuclear cells; n, number of samples; PMN, polymorphonuclear cells. Variables are expressed as mean ± standard deviation when normally distributed and as median (interquartile range) when distribution does not follow normality. ^a^Significant differences between BPE, exudates and BPE, transudates (p<0.05). ^b^Significant differences between total BPE, and total MPE (p<0.05). ^c^Significant differences between NSCLC + ovarian cancer and other malignancies (p<0.05).

In relation to differences within the MPE groups (Table 2), the highest concentrations in PF and serum were observed in the NSCLC + ovarian cancer group. In SCLC and other malignancies, HE4 concentrations were lower even as compared to the transudative BPE group. No statistically significant differences were observed in HE4 ratio between the SCLC + ovarian cancer group and other malignancy group.

Data were displayed on a box plot to assess the influence of eGFR on HE4 levels (Supplementary Figure 1). HE4 levels in PF and serum showed a tendency to increase as eGFR decreased, whereas the opposite tendency was observed in the ratio. The patients with eGFR <30 mL/min/1.73 m^2^ exhibited the highest HE4 levels in PF and serum and the lowest ratio values.

Finally, patients were divided into two groups according to their eGFR, where 223 patients (88.1 %) presented eGFR >30 mL/min/1.73 m^2^, and 30 patients (11.9 %) had an eGFR <30 mL/min/1.73 m^2^.

**Figure 2:**
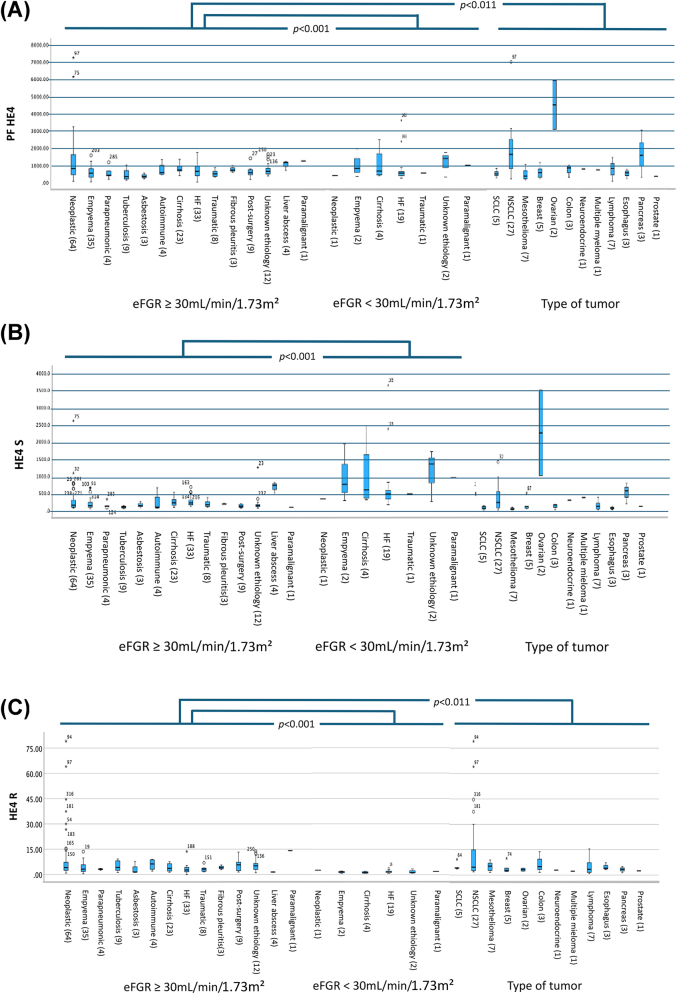
Distribution of HE4 concentrations according to the different etiologies of pleural effusion, based on glomerular filtration rate and the histology of malignant pleural effusion, in pleural fluid samples (A), serum (B), and pleural fluid/serum ratio (C). The number of cases per etiology is shown between brackets. SCLC, small cell lung cancer; NSCLC, non-small cell lung cancer; HF, heart faillure; PF, pleural fluid; eGFR, estimated glomerular filtration rate; R, ratio.

Figure 2 displays HE4 concentration distribution in PF, serum and ratio by eGFR and PE etiology. The patients with eGFR <30 mL/min/1.73 m^2^ exhibited the highest HE4 levels in PF (median 848 vs. 634 pmol/L, p<0.001) and serum (median 541 vs. 153 pmol/L, p<0.001) and a lower ratio (median 1.47 vs. 2.98, p<0.001), as compared to patients with a higher eGFR.

**Figure 3:**
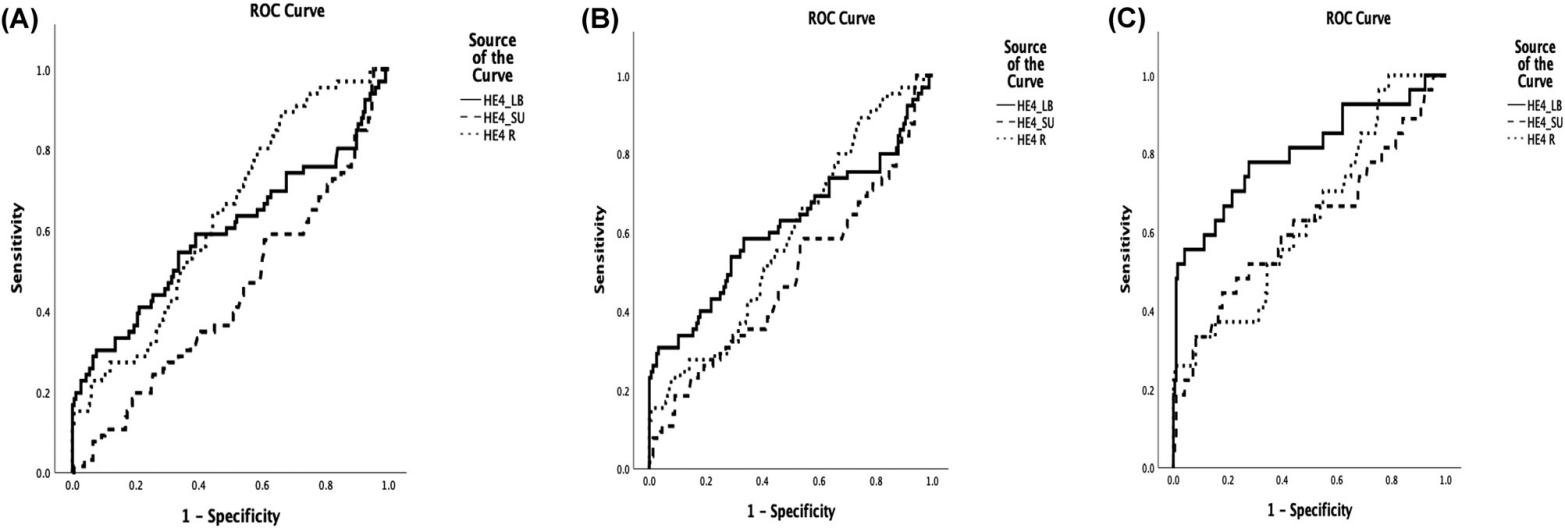
ROC curves for HE4 in pleural fluid, serum and ratio for the diagnosis of malignant pleural effusion. (A) ROC curves for the whole group. (B) ROC curves for patients with eGFR >30 mL/min/1.73 m^2^. (C) ROC curves for patients with eGFR >30 mL/min/1.73 m^2^ for ovarian cancer and NSCLC. LB, pleural fluid; R, ratio; SU, serum.

Table 3 contains the results for diagnostic performance of HE4 for MPE. ROC curves are displayed in Figure 3. ROC curve analysis revealed statistically significant discrimination for HE4 and the ratio (p<0.05), with the ratio yielding the greatest AUC, followed by HE4 in PF. Whereas the DeLong test on ROC curves excluded any statistically significant differences between PF and ratio, differences with serum were significant. In patients with eGFR >30 mL/min/1.73 m^2^, significant differences were noted between the ROC curve for PF and serum, without any differences observed between PF and ratio or between serum and ratio.

**Table 3:** HE4 diagnostic performance for MPE in PF, serum and ratio for all study samples and samples with eGFR ≥30 mL/min/1.73 m^2^.

	Total (n=253)								eGFR ≥30 mL/min/1.73 m^2^ (n=223)					
	AUC	95 % CI	C-off, pmol/L	S	Sp	NPV	PPV	AUC	95 % CI	PC, pmol/L	S	Sp	NPV	PPV
HE4 PF, pmol/L	0.590	(0.500–0.680)	2,291	16.7^a^	100	77.7	100	0.614	(0.522–0.705)	1,778	23.1^c^	100	76.3	100
Serum HE4, pmol/L	0.437	(0.355–0.520)	3,586	0	100	74.3	NA	0.491	(0.403–0.580)	1,923	1.5^d^	100	71.5	100
HE4 ratio	0.632	(0.557–0.707)	14.56	12.1^b^	100	76.7	100	0.584	(0.502–0.666)	14.56	12.3^e^	100	73.8	100

^a^11 MPE: 8 lung, 2 ovarian, 1 pancreas. ^b^8 MPE: 7 lung, 1 hematological. ^c^MPE: 12 lung, 2 ovarian, 1 pancreas. ^d^1 MPE: 1 ovarian. ^e^8 MPE: 7 lung, 1 hematological (8). AUC, area under the curve; Sp, specificity, CI, confidence interval; PF, pleural fluid; n, number of samples; C-Off, cut-off; S, sensitivity; NPV, negative predictive value; PPV, positive predictive value.

Finally, in the NSCLC + serous ovarian cancer group, the AUC of this marker in PF increased to 0.802, thereby suggesting almost exclusive discrimination of this type of tumors. The AUC of HE4 in serum and HE4 ratio increased to 0.619 and 0.639, respectively (Figure 3). Comparative analysis of the ROC curves revealed significant differences between PF vs. serum and vs. ratio, without any significant differences between serum and ratio.

With respect to the applied cut-off points, HE4 demonstrated restricted sensitivity at levels of maximum specificity for both PF and ratio (Table 3). In serum, no cut-off value could be established for the highest specificity. Sensitivity increased when the eGFR ≥30 mL/min/1.73 m^2^ group was selected following cut-off adjustment. Across all selected cut-off values, the majority of detected cases corresponded to lung and ovarian cancer. A case of pancreatic cancer and another case of hematological neoplasm were also identified (Table 3).

## Discussion

This study examines the diagnostic performance of HE4 for MPE. HE4 concentrations in PF were significantly higher in patients with MPE, as compared to patients with BPE. At the cut-off values established, sensitivity was 16.7 % for PF HE4 and 12.1 % for the HE4 ratio, achieving the highest specificity in the two cases (100 %). Following adjustment of cut-off values, sensitivity increased when patients were stratified by eGFR. The group that showed the highest HE4 levels was the NSCLC + ovarian cancer, consistently with the results of previous studies [7], 17], 18].

There are several studies in the literature assessing the diagnostic performance of HE4 for MPE. However, studies are heterogeneous, especially in terms of the types of MPEs investigated. The studies focused on lung cancer, such as those conducted by Demirbas et al. and Lv et al. [18], 24], reported very high AUCs (0.87 and 0.831, respectively), since HE4 is overexpressed in this type of cancer. In contrast, when MPEs are evaluated as a whole by including different types of neoplasms, inconsistent results are obtained concerning the diagnostic performance of HE4, with AUCs ranging from 0.68 to 0.89 [17], 19], 25], 26]. In our study, we obtained an AUC of 0.59, the lowest result reported.

We also examined differences between HE4 levels across the different types of BPE, with higher HE4 concentrations found in transudates, as compared to exudates. Elsammak et al. [19] and Demirbas et al. [24] did not find any differences in HE4 levels between BPE transudates and exudates, which may be explained by the type of benign disease or differences in the number of cases recruited.

There is robust literature on the association between serum HE4 levels and kidney disease [15], 16], [27], [28], [29], [30], with acute and chronic RI being associated with higher HE4 concentrations, even at early stages of the disease [29]. Hertlein et al. [15] and Escudero et al. [30] assessed differences between healthy patients and patients with benign diseases and observed higher HE4 concentrations in patients with RI. In view of the strong influence of RI, HE4 concentrations should be interpreted considering kidney function parameters [29]. Patients undergoing hemodialysis and critically-ill patients were excluded from our study, as they presented confounding factors.

When patients were stratified by eGFR, we observed that HE4 concentrations in PF and serum were higher when eGFR <30 mL/min/1.73 m^2^, as compared to patients with higher eGFRs. Comparable results were reported by Bérgamo et al. in their studies in PF [7] and ascitic fluid [20]. Our study also revealed higher serum HE4 concentrations, which supports the hypothesis of HE4 diffusion from plasma to biological fluids. The influence of eGFR in patients with MPE could not be examined, as only a case of RI was included.

The literature also describes the correlation between HE4 and heart failure severity [27], 28] and identifies HE4 as a prognostic predictor of acute [27], 28] and chronic [28] heart failure. Our results support these findings, demonstrating higher HE4 levels in BPE in patients with heart failure. Likewise, elevated levels were observed in BPE in cirrhotic patients, as reported by other authors [15], 29].

As to the utility of serum HE4 levels as a diagnostic marker of solid neoplasms, two meta-analyses assessing their use for lung cancer diagnosis established cut-off values at 50–149 pmol/L [14], 16]. In contrast, different cut-off values were established in a meta-analysis involving patients with ovarian cancer, with a maximum cut-off value of 150 pmol/L [31]. In our study, BPE patients presented a median of 189 pmol/L, which is significantly higher than the cut-off values previously established in the literature. Therefore, applying the same serum cut-off values for diagnosis of solid neoplasms in patients with PE would not be appropriate, especially in the case of patients with NSCLC and ovarian cancer.

The highest HE4 ratios were observed in MPE patients, as compared to BPE ones; this finding, however, should be interpreted with caution. Patients with transudative BPE, – who generally present other concomitant diseases such as RI, heart failure or cirrhosis – may exhibit higher serum HE4 concentrations than patients with MPE. In some cases, transudative BPE patients may exhibit higher PF HE4 concentrations than patients with neoplasms other than NSCLC or ovarian cancer (see Table 2), yet a lower HE4 ratio. Therefore, calculating the HE4 ratio can be useful to determine whether elevated HE4 levels are secondary to a benign disease, since HE4 ratios are lower in BPE than in MPE. On another note, solid conclusions could not be obtained on differences in the transudative MPE subgroup, since only two cases – one case of breast cancer and another of NSCLC – were included, with ratios of 2.67 and 4.65, respectively.

To date, only Elsammak et al. [19] had explored the utility of the HE4 ratio. The authors found no significant differences between transudativ/exudative BPE and MPE, although kidney function was not considered in that study.

When comparing HE4 ratios with those documented for other tumor markers, our HE4 ratios exceeded the cut-off values previously proposed by other authors (ratio≈1.2 [6], 8]). This finding could be explained by mesothelial secretion of HE4. HE4 patterns would be comparable to those of other secreted markers, like CYFRA 21.1 and CA 125 [8]; hence, the cut-off values used for non-secreted tumor markers would not be applicable. In our study, the cut-off for the ratio with 100 % specificity was established at 14.56, with pulmonary neoplasms showing the highest ratios. None of the cases of ovarian cancer were detected at the cut-off established, as they presented ratios of 2.35 (concentrations of 6126 pmol/L and 2605 pmol/L in PF and serum, respectively) and 3.99 (concentrations of 3178 pmol/L and 797 pmol/L in PF and serum), respectively. Plasma HE4 concentrations were very elevated in these cases due to HE4 production from metastatic ovarian tumors. This differentiates it from other neoplasms, which have mean and median plasma concentrations of 207 and 151 pmol/L, respectively. Elevated ratios of 13–14 were observed in several BPE cases (like heart failure, empyemas, pancreatitis and postsurgical processes). These cases underline the key role of identifying the presence of very elevated HE4 levels in benign diseases, approaching each case on a case-by-case basis and interpreting results in the specific clinical context of each patient.

In PF, the HE4 cut-off of 2291 pmol/L demonstrated a sensitivity of 16.7 % with 100 % specificity. The cut-off obtained was comparable to the one reported by Bérgamo et al. [7], who described a discriminative value of 3050 pmol/L in PF with a sensitivity of 22.9 and 100 % specificity. Due to the influence of RI on HE4 values, cut-off values were adjusted to eGFR ≥30 mL/min/1.73 m^2^. We found that a lower cut-off value in PF (1778 pmol/L) had higher sensitivity (23.1 %). Bérgamo et al. [7] also defined a lower cut-off for patients with eGFR ≥30 mL/min/1.73 m^2^, set at 1992 pmol/L (sensitivity of 28.6 and 100 % specificity). Similar AUC values were documented for HE4 in PF, being 0.590 in our study and 0.692 in the study by Bérgamo [7]. In both studies diagnostic performance improved in patients with eGFR ≥30 mL/min/1.73 m^2^, with AUCs of 0.614 and 0.722, respectively. The differences observed between the two studies could be due to the lower proportion of gynecological neoplasms included in our series (only two cases).

Limitations of this study include those inherent to single-centre studies, which may limit the applicability of our results to other populations. On another note, in the MPE group, some tumor histologies were underrepresented, such as gynecological cancer and SCLC, which hindered appropriate analysis of differences between NSCLC and SCLC. In addition, the limited presence of patients with MPE and eGFR <30 mL/min/1.73 m^2^ prevented us from assessing the impact of RI on HE4 levels in this subgroup.

In conclusion, HE4 is a widely used marker of ovarian cancer than may also be useful in PEs. Elevated HE4 levels may identify MPE with 100 % specificity and provide guidance on the origin of the primary tumor, with higher levels corresponding to ovarian and lung cancer. In a few hours, HE4 levels may suggest the presence of an underlying neoplastic process and support the performance of a diagnostic invasive procedure when cytology is negative. Considering the results of this study, the same serum HE4 cut-off values should not be used in patients with PE, congestive heart failure, cirrhosis or RI as in patients without these conditions, in order to avoid misdiagnosis when interpreting this tumor marker.

## Supplementary Material

Supplementary Material

## References

[1] Beaudoin S, Gonzalez AV (2018). Evaluation of the patient with pleural effusion. Can Med Assoc J.

[2] Villena Garrido V, Cases Viedma E, Fernández Villar A, De Pablo Gafas A, Pérez Rodríguez E, Porcel Pérez JM (2014). Normativa sobre el diagnóstico y tratamiento del derrame pleural. Actualización. Arch Bronconeumol.

[3] Zheng WQ, Hu ZD (2023). Pleural fluid biochemical analysis: the past, present and future. Clin Chem Lab Med.

[4] Volarić D, Flego V, Žauhar G, Bulat-Kardum L (2018). Diagnostic value of tumour markers in pleural effusions. Biochem Med.

[5] Gayen S (2022). Malignant pleural effusion: presentation, diagnosis, and management. Am J Med.

[6] Trapé J, Sant F, Franquesa J, Montesinos J, Arnau A, Sala M (2017). Evaluation of two strategies for the interpretation of tumour markers in pleural effusions. Respir Res.

[7] Bérgamo S, Trapé J, González-García L, González-Fernández C, Vergara C, la-Torre ND (2025). The diagnostic accuracy of HE4 in the differential diagnosis of pleural effusions. Clin Chim Acta.

[8] Trapé J, Bérgamo S, González-Garcia L, González-Fernández C (2024). Lung cancer tumor markers in serous effusions and other body fluids. Tumor Biol.

[9] Yang Q, Niu Y, Zhou Q, Yang DN, Zhu HZ, Yan C (2025). Influences of age and sex on the diagnostic accuracy of human epididymis secretory protein 4 for malignant pleural effusion. Sci Rep.

[10] Heffner JE (2008). Diagnosis and management of malignant pleural effusions. Respirology.

[11] Herrera LS, Fernández-Fabrellas E, Juan Samper G, Marco Buades J, Andreu Lapiedra R, Pinilla Moreno A (2017). Predicting malignant and paramalignant pleural effusions by combining clinical, radiological and pleural fluid analytical parameters. Lung.

[12] Kassirian S, Hinton SN, Cuninghame S, Chaudhary R, Iansavitchene A, Amjadi K (2023). Diagnostic sensitivity of pleural fluid cytology in malignant pleural effusions: systematic review and meta-analysis. Thorax.

[13] Zhong H, Qian Y, Fang S, Yang L, Li L, Gu W (2017). HE4 expression in lung cancer, a meta-analysis. Clin Chim Acta.

[14] Cheng D, Sun Y, He H (2015). The diagnostic accuracy of HE4 in lung cancer: a meta-analysis. Dis Markers.

[15] Hertlein L, Stieber P, Kirschenhofer A, Fürst S, Mayr D, Hofmann K (2012). Human epididymis protein 4 (HE4) in benign and malignant diseases. Clin Chem Lab Med.

[16] Karlsen NS, Karlsen MA, Høgdall CK, Høgdall EV (2014). HE4 tissue expression and serum HE4 levels in healthy individuals and patients with benign or malignant tumors: a systematic review. Cancer Epidemiol Biomarkers Prev.

[17] Yang Q, Niu Y, Wen JX, Yang DN, Han YL, Wen XH (2023). Value of human epididymis secretory protein 4 in differentiating malignant from benign pleural effusion: an analysis of two cohorts. Ther Adv Respir Dis.

[18] Lv M, Wang F, Wang X, Zhang C (2019). Diagnostic value of human epididymis protein 4 in malignant pleural effusion in lung cancer. Cancer Biomark.

[19] Elsammak MY, Attia A, Hassan HA, Zaytoun TM, Shorman M, Suleman M (2012). Evaluation of pleural fluid human epididymis 4 (HE4) as a marker of malignant pleural effusion. Tumor Biol.

[20] Bérgamo S, Trapé J, González-García L, González-Fernández C, Vergara C, de-la-Torre N (2023). Utility of human epididymis protein 4 in the differential diagnosis of ascites. Clin Biochem.

[21] Nuez-Zaragoza E, Bhambi-Blanco I, Vidal-Pla M, Aparicio-Calvente I, Escoda-Giralt MR, Gallardo-Campos J (2025). Utility of the combination of high fluorescence cells and tumor markers for the diagnosis of malignant pleural effusions. Clin Biochem.

[22] Light RW (2013). The light criteria. Clin Chest Med.

[23] Levey AS, Stevens LA, Schmid CH, Zhang YL, Castro AF, Feldman HI (2009). CKD-EPI (Chronic Kidney Disease Epidemiology Collaboration). A new equation to estimate glomerular filtration rate. Ann Intern Med.

[24] Demirbas S, Yerlikaya FH, Yosunkaya S, Can U, Celalettin K (2022). The investigation of levels of endothelial cell-specific molecule, progranuline, clusterin, and human epididymis protein 4 in the differential diagnosis of malignant pleural effusions. Medicine (Baltim).

[25] Li J, Lin L, Yang S, Mu Y, Zhang L, Ruan H (2025). Diagnostic value of CEACAM6 and HE4 in pleural fluid for malignant pleural effusion. Ann Med.

[26] Porcel JM, Esquerda A, Bielsa S, Novell A, Sorolla MA, Gatius S (2019). Epithelial cell adhesion molecule (EpCAM) from pleural fluid cell lysates is a highly accurate diagnostic biomarker of adenocarcinomatous effusions. Respirology.

[27] De Boer RA, Cao Q, Postmus D, Damman K, Voors AA, Jaarsma T (2013). The WAP four-disulfide core domain protein HE4: a novel biomarker for heart failure. JACC Heart Fail.

[28] Piek A, Meijers WC, Schroten NF, Gansevoort RT, De Boer RA, Silljé HHW (2017). HE4 serum levels are associated with heart failure severity in patients with chronic heart failure. J Card Fail.

[29] Kappelmayer J, Antal-Szalmás P, Nagy B (2015). Human epididymis protein 4 (HE4) in laboratory medicine and an algorithm in renal disorders. Clin Chim Acta.

[30] Escudero JM, Auge JM, Filella X, Torne A, Pahisa J, Molina R (2011). Comparison of Serum Human Epididymis protein 4 with cancer antigen 125 as a tumor marker in patients with malignant and nonmalignant diseases. Clin Chem.

[31] Huang J, Chen J, Huang Q (2018). Diagnostic value of HE4 in ovarian cancer: a meta-analysis. Eur J Obstet Gynecol Reprod Biol.

