# Supplementary material for: Determination of HE4 in pleural fluid and ratio: considerations and diagnostic performance for malignant pleural effusion
Source: Adv Lab Med. 2026 Apr 7;7(2):105–15. doi: 10.1515/almed-2025-0175 (PMC13169826; doi:10.1515/almed-2025-0175)
Supplement: Supplementary file 1 — Supplementary Material [file j_almed-2025-0175_suppl_001.docx]

Supplementary Figure 1. Distribution of HE4 concentrations in pleural fluid, serum, and ratio by estimated glomerular filtrate.


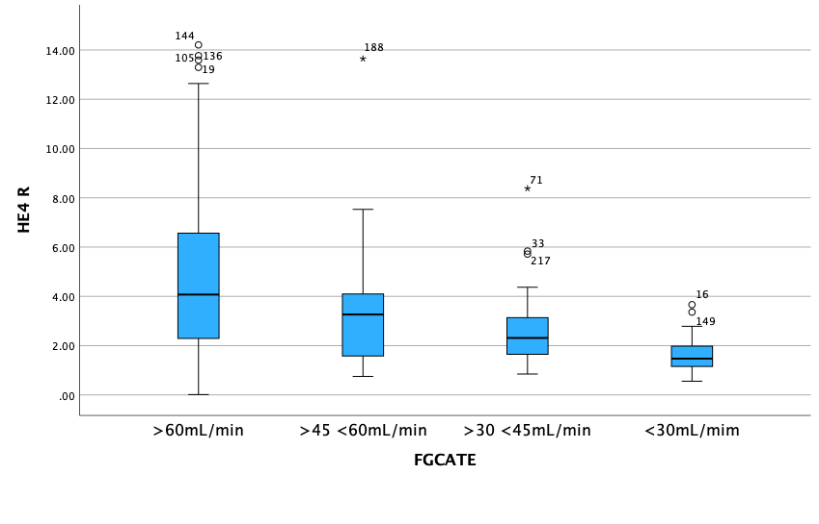

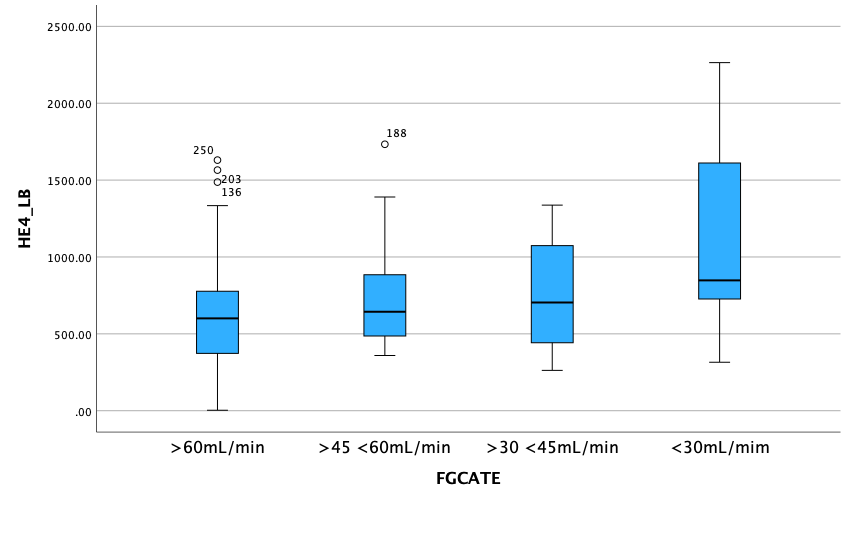

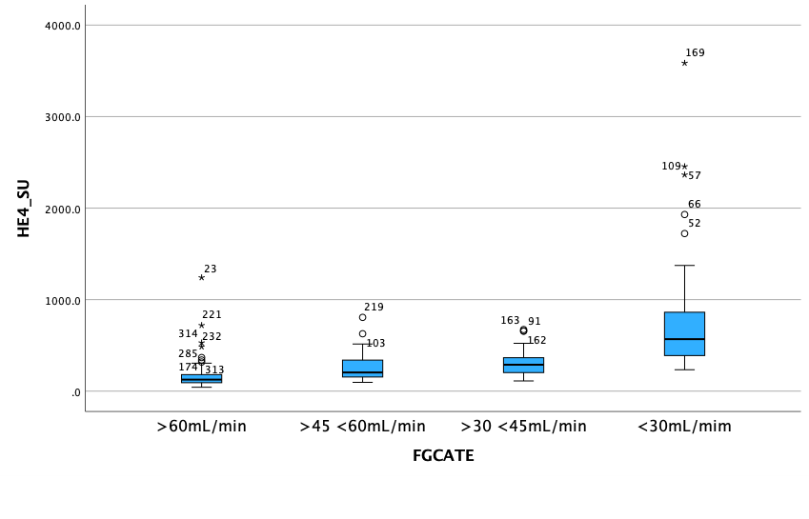


**PF HE4**

**GF**

**GF**

**GF**

**SERUM HE4**

**HE4 RATIO**

*Significant differences (p<0.05) were observed between HE4 concentrations in patients with eGF<30mL/min/1.73m^2^ and other eGF values in pleural fluid, serum and ratio. Median comparison by Mann-Withney U test for all groups. GF: glomerular filtrate; PF: pleural fluid.
